# Overexpression of E2F1 Promotes Tumor Malignancy And Correlates with TNM Stages in Clear Cell Renal Cell Carcinoma

**DOI:** 10.1371/journal.pone.0073436

**Published:** 2013-09-04

**Authors:** Xin Ma, Yu Gao, Yang Fan, Dong Ni, Yu Zhang, Weihao Chen, Peng Zhang, Erlin Song, Qingbo Huang, Qing Ai, Hongzhao Li, Baojun Wang, Tao Zheng, Taoping Shi, Xu Zhang

**Affiliations:** Department of Urology/State Key Laboratory of Kidney Diseases, Chinese PLA General Hospital/Chinese PLA Medical Academy, Beijing, P. R. China; Sapporo Medical University, Japan

## Abstract

**Background:**

Transcription factor E2F1 exerts effects on many types of cancers. As an upstream regulator of a host of genes, E2F1 can trigger diverse aberrant transcription processes that may dominate malignancy. Clear cell renal cell carcinoma (ccRCC) is the most common subtype in renal cell carcinoma which displays high malignancy and has a shortage of biomarkers in clinics. Our study aimed to explore the function of E2F1 in ccRCC and its correlation with clinicopathological parameters.

**Methodology/Principle Findings:**

Transcription factor E2F1 was mainly distributed in cancer cell nucleus and mRNA expression signiﬁcantly increased in 72 cases of clear cell renal cell carcinoma (ccRCC) tissues compared with adjacent non-cancerous kidney tissues (*p*<0.001). The protein expression was consistent with mRNA expression. Further analysis in 92 cases indicated that E2F1 mRNA level expression was associated with the tumor pathologic parameters embracing diameter, Fuhrman tumor grade, pT stage, TNM stage grouping and macrovascular infiltration (MAVI). These surgical specimens had high grade tumors accompanied with an elevated E2F1 expression. Moreover, E2F1 transfection was found to contribute significantly to cancer cell proliferation, migration and invasion in vitro.

**Conclusions/Significance:**

Overexpression of E2F1 may be a key event in the local and vascular infiltration of ccRCC indicated by the activation of matrix metalloproteinase (MMP) 2 and MMP9. These findings highlighted the implication of E2F1’s function in the metastatic process. Furthermore, the clinical relevance of E2F1 in ccRCC pointed to a potential new therapeutic target.

## Introduction

Renal cell carcinoma (RCC) is the second leading cause of death in urological malignant neoplasms, accounting for 2% to 3% of all adult malignant diseases [1]. The five major subtypes of RCCs are clear cell, papillary, chromophobe, collecting duct, and unclassified types. The most common subtype is clear cell renal cell carcinoma (ccRCC) [2]. Few biomarkers for ccRCC have been demonstrated for clinical diagnosis and outcome prediction, although several molecules have been suggested as potential solutions. For instance, mutation of the von Hipple–Lindau tumor suppressor gene has been proven to be a hallmark of ccRCC [3]. It is known that invasion of adjacent larger blood vessels is of high malignancy, signifying a pro-metastatic state [4]. However, the mechanism of tumor progression and vascular infiltration of ccRCC is poorly understood, and thus requires research on carcinogenesis and cancer development.

E2Fs comprise a family of eight transcription factors that can be classified into different groups based on domain conservation and transcriptional activity. E2F1 is the first cloned member and plays an imperative role in cell fate control. E2F1 also functions as a starting switch in complex crossways binding to promoters of downstream genes [5]. Numerous studies have reported that E2F1 expression was of clinical significance in different cancers [6–10]. Attributing to its multiple modulations and upper regulatory effects, alterations were to be explored in ccRCC to the best of our knowledge. A logical linking was established between E2F1 mRNA level expressions and specimen pathological parameters by our study. As expected, in vitro assay showed that E2F1 overexpression in ccRCC cell line 786-O and A498 boosted cancer cell growth and promoted the cell invasive capacity. Meanwhile, E2F1 knockdown in Caki-1 did the opposite. For further investigation we explored the expression of MMP2 and MMP9, cell-surface proteolysis of extra-cellular matrix components, which may exert crucial effect in late stage carcinogenesis and vascular invasion of ccRCC.

## Materials and Methods

### Ethics Statement

Written informed consent for a tumor-oriented study was obtained from all patients prior to sample collection and the study was approved by the Protection of Human Subjects Committee of Chinese People’s Liberation Army General Hospital.

### Sample collection and analysis

We retrospectively evaluated data on 92 patients being admitted at the Department of Urology in Chinese PLA General Hospital in 2009. These patients were all operated and seventy-two pairs of RCC tissues and their corresponding adjacent non-cancerous kidney tissues were collected with RCC tissues having been clinically and pathologically conﬁrmed to be of the clear cell type. All RCC specimens were staged according to the 2011 Union for International Cancer Control (UICC) TNM classification of malignant tumors. The Fuhrman nuclear grading system was used to determine the nuclear grade. Macrovascular invasion (MAVI) displayed renal vein or inferior vena cava invasion which signified tumor malignancy. The specimens were immediately snap-frozen in liquid nitrogen after resection. They were stored at -80°C until analysis.

### Cell culture and reagents

The ccRCC cell lines, 786-O, A498, Caki-1, Caki-2 as well as the human renal proximal tubular epithelial cell line HKC [11], were obtained from our laboratory. The cells were cultivated in RPMI 1640 medium/DMEM-F12 and Dulbecco’s modiﬁed Eagle’s medium (Gibco, Gran Island, NY), with penicillin (100 U/ml), and streptomycin (100 U/ml) respectively, supplemented with 10% fetal calf serum in a humidiﬁed incubator at 37°C with a mixture of 95% air and 5% CO_2_.

### RNA isolation and real-time PCR

The total cellular RNA of tissues and cell lines were extracted using Trizol reagent (Invitrogen, Carlsbad, CA) and were reversely transcribed to cDNA using one-step RT-PCR kit (TransGen Biotech Co., Ltd, Beijing, China) according to the manufacturer’s instructions. Quantiﬁcation of gene expression was performed using the ABI PRISM 7500 Sequence Detection System (Applied Biosystems, Foster City, CA) with SYBR Green (TransGen Biotech Co., Ltd, Beijing, China). The relative mRNA levels of E2F1 were normalized to peptidylprolyl isomerase A (PPIA) [12] using the 2^-ΔΔCT^ method. The primer sequences were given in Table 1. The experiments were repeated 3 times and every sample was performed in triplicate.

**Table 1:** Real-time PCR Primers.

Gene	Primer Sequence	Amplicon
E2F1	Forward primer: CAGAGCAGATGGTTATGG(18bp)	77bp
	Reverse primer: CTGAAAGTTCTCCGAAGA(18bp)	
PPIA	Forward primer: ATGGTCAACCCCACCGTGT(19bp)	101bp
	Reverse primer: TCTGCTGTCTTTGGGACCTTGTC(23bp)	
MMP2	Forward primer: GCGGCGGTCACAGCTACTT(19bp)	71bp
	Reverse primer: CACGCTCTTCAGACTTTGGTTCT(23bp)	
MMP9	Forward primer: CCTGGAGACCTGAGAACCAATC(22bp)	80bp
	Reverse primer: CCACCCGAGTGTAACCATAGC(21bp)	
vimentin	Forward primer: ACTACGTCCACCCGCACCTA(20bp)	171bp
	Reverse primer: CAGCGAGAAGTCCACCGAGT(20bp)	
ZEB1	Forward primer: AGAGATGACTTGTTATAGCA(20bp)	160bp
	Reverse primer: AATTTGTTTCTACCACAGTATT(22bp)	

### Western blot assay

Total protein of the tumor cells was obtained using RIPA lysis buffer (Santa Cruz) containing a mixture with proteinase inhibitors (Roche Applied Science). BCA reagent (Applygen Technologies) was used to quantify protein amount. Equivalent amounts of protein (30-50 µg) were denatured and separated by SDS-polyacrylamide gels (SDS-PAGE), thereafter being transferred to PVDF membranes followed by one hour non-fat milk blocking. Blots were then incubated with primary antibody anti-E2F1 at 4°C overnight, washed three times with TBST solution and later incubated with the corresponding secondary antibody for one hour at room temperature [13]. E2F1 primary antibody was purchased from Abcam Biotechnology. Proteins were rabbit anti-E2F1 (Abcam, Cambridge, MA) at a dilution of 1:1000 ,mouse anti-GAPDH (ZSGB-BIO) at a dilution of 1:1000 and mouse anti-β-actin (ZSGB-BIO) at a dilution of 1:1000. In all specimens, goat anti-mouse IgG-HRP and goat anti-rabbit IgG-HRP (ZSGB-BIO) were used as the secondary antibody at a dilution of 1:3000 respectively. The membranes were processed using the Super Signal West Maximum Sensitivity Substrate Kit (Pierce, Rockford, IL) and exposed to X-ray film (Kodak, Rochester, NY).

### Plasmid transfection

Cell lines 786-O and A498 were used for transfection. Cells were seeded into 60 mm plates (2×10^5^ cells/well, 1×10^5^cells/well for 786-O alone) 24 hours prior to plasmid transfection. In cell lines E2F1 was over-expressed using HA-tagged E2F1 plasmid by *Polyplus*-transfection agent (Illkirch, France), with the empty vector (pcDNA3.1-entry) as control. HA-tagged E2F1 plasmid was a kind help from Professors Mian Wu [14]. The cells were harvested at indicated time points.

### RNAi knockdown

The small interfering RNA (siRNA) targeting E2F1 was designed with sequence Sense: 5=-GCGCAUCUAUGACAUCACCTT-3=; Anti-sense: 5=-GGUGAUGUCAUAGAUGCGCTT-3=, for negative control (Sense: 5=-UUCUCCGAACGUGUCACGUTT-3=; Anti-sense: 5=-ACGUGACACGUUCGGAGAATT-3=), they were designed and synthesized by Shanghai Gene-Pharma Co. (Shanghai, China). For the RNAi knockdown, equal cell amount in two groups were seeded in the plates containing medium without antibiotics for 24 h prior to the transfection. The siRNAs were transfected into the cells using Lipofectamine 2000 in serum-free Opti-MEM, according to the manufacturer’s instructions. The expression levels of E2F1 were determined by real-time PCR and western blot analysis. The cells were harvested at the indicated time points and used for further analysis.

### Immunohistochemistry

Sequential sections (4-µm thick) from human ccRCC and noncancerous kidney tissues were cut from the parafﬁn blocks. Slides were deparafﬁnized and dehydrated in gradient alcohol, dimethylbenzene for 20 min twice, dipped into 100% alcohol for 10 min twice, and then sequentially immersed in 80% and 70% alcohol for 5 min respectively. For antigen repair, sections were dipped in citric acid buffer at 95 °C for 20 min, cooled down to room temperature and incubated with 3% H_2_O_2_ in methanol for 30 min at 37°C and blocked with normal goat serum 30 min at 37°C. After that these sections were incubated with the indicated primary antibodies overnight at 4°C according to standard procedures. Primary antibodies were rabbit polyclonal E2F1(Abcam, Cambridge, MA) (1:100 dilution), after washing in PBS, the sections were treated with biotin-labeled serum (1:200) for 30 min, rinsed with PBS, and visualized by Envision kit/HRP (DAB) (ZSGB-BIO, China) (DAB). Thus retrograde alcohol dealing (80%,95%,100%,100%) for 2min consecutively, Sections were counterstained in hematoxylin and mounted in Permount, afterwards for microscopic evaluation. Negative control was performed by replacing the primary antibody with rabbit serum. All slides were examined and scored independently by three pathological consultants without knowing patients’ clinical data E2F1 expression was evaluated according to the ratio of positive cells per specimen quantitatively and scored 0 for staining <1%, 1 for staining of 2% to 25%, 2 for staining of 26% to 50%, 3 for staining of 51% to 75%, and 4 for staining >75% of the cells counted. Staining intensity was defined as follows: 0, no signal; 1, weak; and 2 for strong staining. A total score of 0 to 8 was ﬁnally calculated and graded as negative (-; score: 0–1), weak (+; 2–4), and strong (++; 5–8) [15].

### Cell proliferation assay

Cells were seeded into 60 mm plates (1×10^5^ cells/well for 786-O and 2×10^5^ cells/well for A498) 24 hours prior to transfection. The cells were then transfected with plasmids as indicated above, and 1000 cells/well were reseeded into 96-well plates and incubated at different time points (0, 24, 48, 72, and 96 h). The effect of E2F1 knockdown in Caki-1 was examined. Thereafter 20µl of MTS (CellTiter 96 AQueous One Solution Reagent; Promega, Madison, WI) was directly added to the cultured wells, which were incubated for 4h. The absorbance at 490 nm was recorded with a 96-well plate reader.

### Plate colony-forming assay

Prepared 786-O and A498 cells were harvested after 48 h HA-tagged E2F1 plasmid transfection and were plated in 6-well plate (500 cells/well), with the two negative controls of empty and pcDNA3.1 entry. Cells were tenderly detached in each well. The number of foci >100 cells was counted after 14 days. Each experiment was performed in triplicate

### Cell cycle analysis

24 h prior to transfection, cells were seeded into 60 mm plates (2×10^5^ cells/well for A498 and 1×10^5^ cells/well for 786-O). The cells were then transfected with plasmids as indicated above and fixed in 70% ice-cold ethanol overnight. Cells subjected to cell cycle assays were identiﬁed by propidium iodide (PI) staining (Beyotime, Shanghai, China) 24 h later in accordance with the manufacturer’s instructions. For RNAi knockdown, Caki-1 cells transfected with siRNA-E2F1 and control sequence were also used for cell cycle analysis. DNA analysis was performed using a BD FACSCalibur (Becton-Dickinson, Franklin Lakes, NJ) ﬂow cytometer. Each experiment was performed in triplicate and repeated three times.

### Cell migration and invasion assay

For cell invasion assay, Boyden chambers containing Transwell (Corning Costar Corp., Cambridge, MA) membrane filters with pore size of 8 µm were used. 60µl Matrigel diluted in optimum was coated in Invasion Chambers rehydrated by adding 0.5 ml RPMI-1640 medium to the upper chambers for 2h at 37 °C overnight. After rehydration, the medium was carefully removed, and 2×10^4^ cells were added to the upper chambers in triplicate. The lower compartments of migration and invasion chambers were ﬁlled with medium of DMEM-F12 containing 1% bovine serum albumin (Sigma) at concentration of 5% and 20% respectively, 4 h or 8 h later, non-migrating cells on the upper surface of the filters were swept by cotton swabs. The migrated and invaded cells on the lower surface of membrane were fixed and stained with methanol mixed crystal violet. Cells were counted under microscope. All assays were run independently three times.

### Wound healing assay

For wound healing assay, cell lines 786-O, A498 and Caki-1 were seeded on 6-well plates with fresh medium containing 1% FBS. Conﬂuent monolayer cells were scraped with a plastic 200 µl pipette tip. After replacing the cell culture medium, the wound closure was photographed at different time points (0, 12, and 24h after scraping). Under 100 × magnifications, the coverage of scraping area was measured by viable cells migrating from both sides. Experiments were performed in triplicate.

### Statistical analysis

All data were analyzed using the SPSS statistical software 13.0(SPSS Inc., Chicago, IL), and *p*<0.05 was considered statistically signiﬁcant. ANOVA and Student’s *t*-test were used as appropriate for all results. Spearman rank correlation analysis was used to determine the correlation of the expression level of E2F1 and clinicopathological parameters, the E2F1 expression was shown as ΔCT mean ± standard deviation (SD).

## Results

### Abundant expression of E2F1 in ccRCC samples versus corresponding noncancerous tissues.

**Figure 1:**
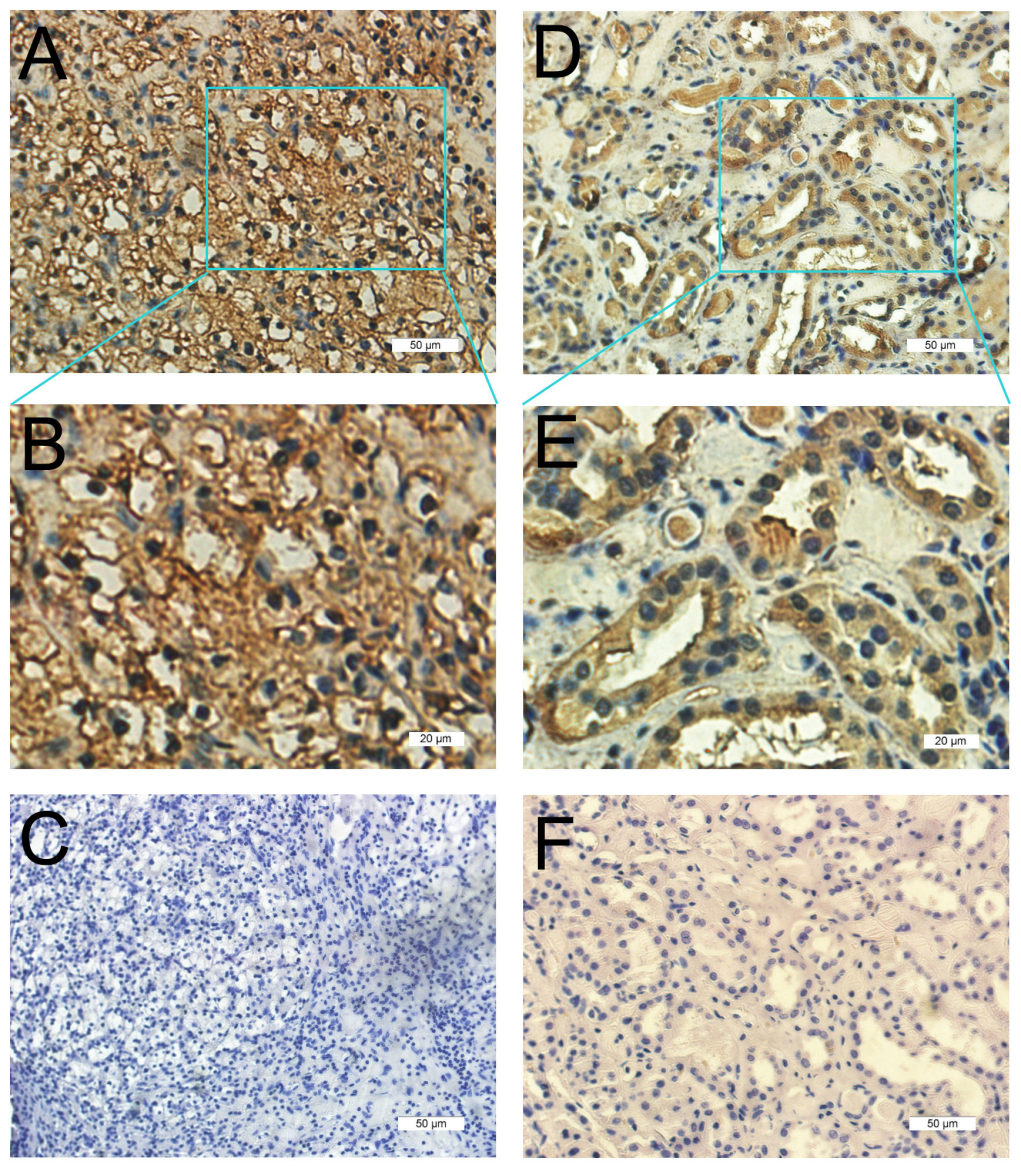
**ccRCC** tissues(38 cases) and their corresponding adjacent non-cancerous kidney tissues were immunohistochemically stained by E2F1 antibody(1:100). Three representative photographs were taken at different magnifications in ccRCC tissues(A-B: 200× A, 400× B and negative control of tumor cells of C) and corresponding adjacent non-cancerous kidney tissues(D-E:200× D,400×E and negative control of normal renal tubules cells of F) respectively.

The expression pattern of E2F1 was evaluated by immunohistochemistry in 38 pairs of ccRCC samples and corresponding noncancerous tissues. Results showed that E2F1 was mainly distributed in the cytoplasm and membrane of renal proximal tubular cells from adjacent noncancerous samples, by contrast, in ccRCC tissues nucleus was predominantly immunohistochemically stained. As shown in Table 2, E2F1 positive expression in ccRCC was 92.11% (35/38), which was significantly higher than 60.53% (23/38) in the peritumoral tissues(*p*<0.05) (Figure 1).

**Table 2:** 

	Number of cases	E2F1 immunostaining		
		-	+	++
ccRCC	38	3 (7.89%)	19 (50%)	16 (42.11%)
Peritumoral tissue	38	15 (39.47%)	15 (39.47%)	8 (21.06%)

Note. Interpretation of E2F1 staining was described in Section 2. E2F1 staining was graded as negative (- score: 0–1), weak (+ 2–4), and strong (++ 5–8).

### Up regulation of E2F1 in ccRCC tissues and ccRCC-derived cell lines.

**Figure 2:**
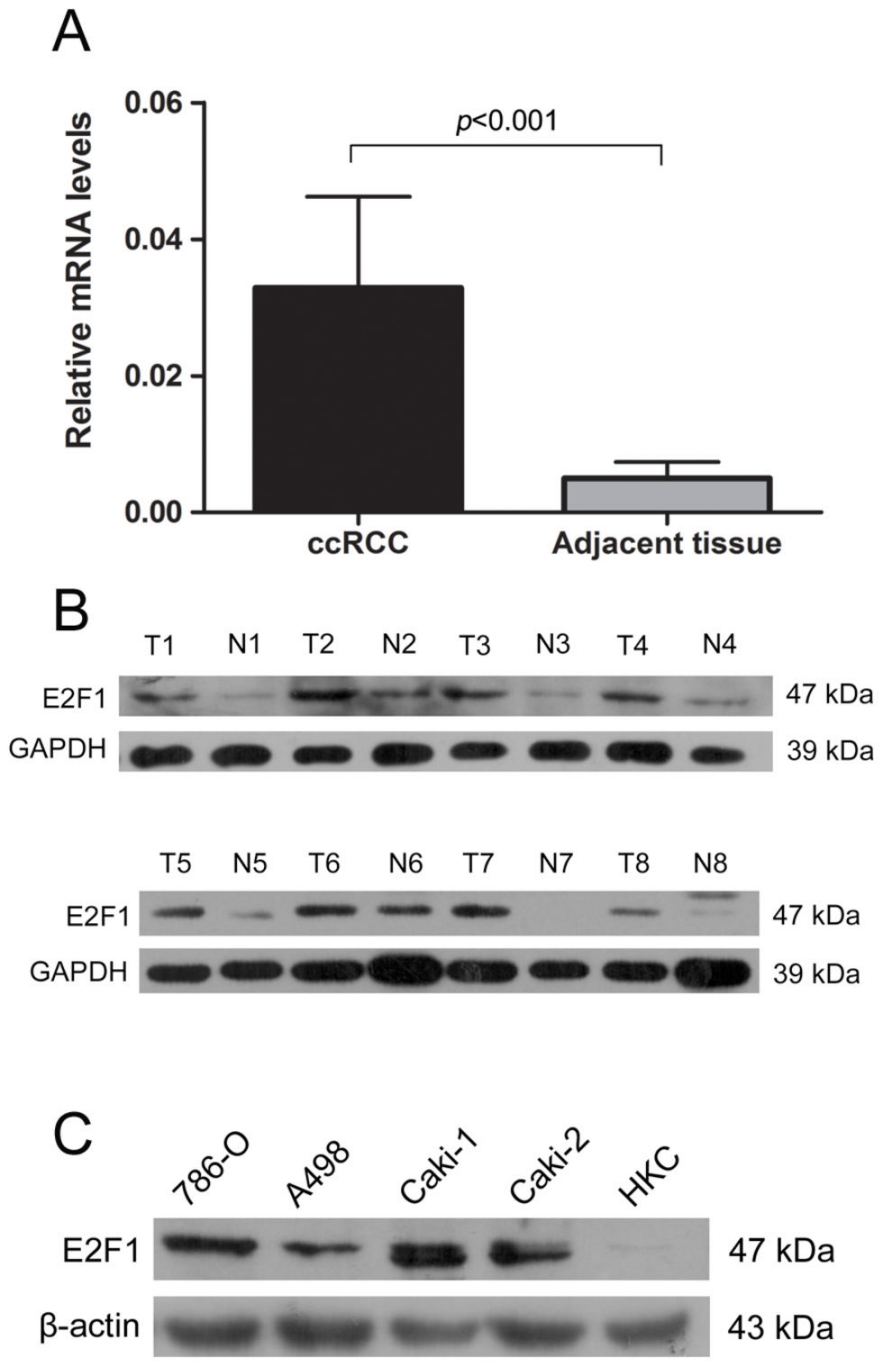
Comparison of mRNA and protein levels expression of E2F1 in **ccRCC** tissues and their corresponding adjacent non-cancerous kidney tissues. (A) Result of E2F1 mRNA expression difference in tumor group and normal renal tissue group(*p*<0.001). (B) Western blot analysis of the consisted proteins of E2F1 in tumor group (T) relative to normal renal tubule cell group (N). Glyceraldehyde-3-phosphate dehydrogenase (GAPDH) was used as internal control for equal loading of samples. (C) Expressions of E2F1 protein in ccRCC cell lines and human renal tubule cell line (HKC) with the control ofβ-actin.

Real-time PCR was performed to explore the mRNA levels of E2F1 in 72 pairs surgical samples. E2F1 expression was significantly higher in tumor group than in noncancerous tissue group (*p*<0.001) (Figure 2A). Analysis on the relationship of E2F1 mRNA expression and clinicopathological parameters in patients with ccRCC (92 cases) was presented in Table 3. E2F1 mRNA levels were significantly associated with tumor diameter(*p*=0.001) (*r*=0.265, *p*=0.011), Fuhrman tumor grade(*p*=0.001) (*r*=-0.366, *p*<0.001), pTstage(*p*=0.003) (*r*=-0.330, *p*=0.001), TNM stage grouping(*p*=0.001) (*r*=-0.363, *p*<0.001) and MAVI(*p*=0.001) (*r*=-0.377, *p*<0.001), but not age and sex. Western blot was performed to determine whether protein levels of E2F1 were consistent with mRNA levels. As expected, the alterations in the protein levels were in agreement with the changes in mRNA levels (Figure 2B). As shown in Figure 2C, ccRCC-derived cell line 786-O, A498, Caki-1, and Caki-2 had a drastic increase of E2F1 protein expression versus the normal renal proximal tubular cell line HKC, revealing metastatic ccRCC cell line Caki-1 the highest of all.

**Table 3:** The relationship of E2F1 (mRNA) with the clinicopathological features in patients with **ccRCC** .

Clinicopathological features	N	E2F1	*p* value
		(ΔCT mean± SD)	
Age(years)			0.230
<40	12	8.64950±3.09432	
≥40;<60	64	8.82904±3.55734	
≥60	16	10.55430±4.58941	
Sex			0.267
Male	61	8.79698±3.80192	
Female	31	9.71307±3.53531	
Tumor diameter(cm)			0.001
≤4	46	9.81429±3.13459	
>4;≤7	30	9.70844±3.93873	
>7	16	5.93818±3.39513	
		*r*=0.265	0.011
Fuhrman tumor grade			0.001
G1	71	9.80366±3.51073	
G2	13	7.65630±3.90117	
G3	8	5.26621±2.21209	
		*r*=-0.366	<0.001
pT stage			0.003
pT1	74	9.72789±3.45790	
pT2	5	7.62008±4.25760	
pT3+ pT4	13	6.13516±3.64816	
		*r*=-0.330	0.001
TNM stage grouping			0.001
Stage I	73	9.76471±3.46720	
Stage II	5	7.62008±4.25760	
Stage III	7	8.19460±3.51311	
Stage IV	7	4.20499±2.26230	
		*r*=-0.363	<0.001
MAVI			<0.001
Macrovascular invasion	12	5.47568±2.88983	
No macrovascular invasion	80	9.65016±3.53245	
		*r*=-0.377	<0.001

### Overexpression of E2F1 protein promoted the proliferation of ccRCC cell lines and enhanced the number of colony forming.

**Figure 3:**
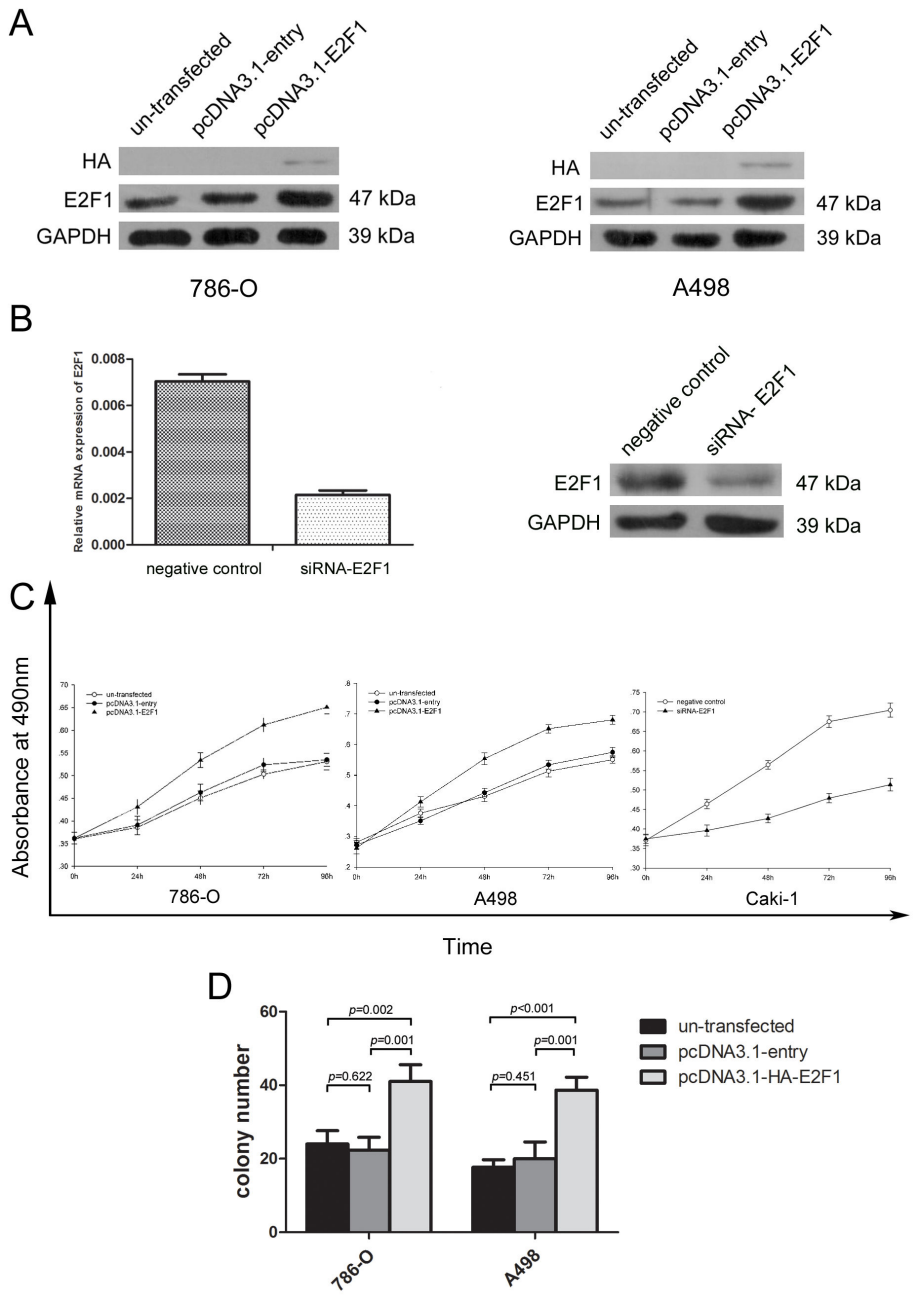
Overexpression of E2F1 led to the proliferation of the tumor cells in vitro. (A) Alteration in the protein levels of the 786–0 and A498 cells 48 hours after transfection with entry or E2F1 plasmid (B). The expression levels of E2F1 knockdown in Caki-1 were determined by real-time PCR and western blot analysis. (C) MTS assay showed E2F1 overexpression accelerated the proliferation velocity in 786-O and A498 cells and knockdown of E2F1 in Caki-1 decreased the proliferation. Each experiment was performed in triplicate. (D) Effect of E2F1 in colony formation of 786-O and A498 cells. The number of foci >100 cells was counted after 14 days. Each experiment was performed in triplicate.

To validate whether the expression of E2F1 signified the malignancy of ccRCC cells, E2F1 was overexpressed by transfection of pcDNA3.1-HA-E2F1 into ccRCC cell line 786-O and A498. Compared with un-transfected group and pcDNA3.1-entry group, the protein level of E2F1 in pcDNA3.1-E2F1 group was drastically elevated 48 h after transfection (Figure 3A). The expression levels of E2F1 knockdown were determined by real-time PCR and western blot analysis (Figure 3B). As shown in Figure 3C of MTS assay, the growth curves demonstrated that strengthened expression of E2F1 signiﬁcantly increased 786-O and A498 cell growth (*p*<0.05), in contrast, knockdown of E2F1 attenuated cell proliferation in Caki-1 cells. Results of colony formation assay illustrated that compared with the empty and entry groups, pcDNA3.1-E2F1 transfected tumor cells gained the ability to improve the colony forming process(*p*<0.001) in 786-O and A498 cell lines(Figure 3D).

### E2F1 accelerated G1/S transition in cell cycle.

**Figure 4:**
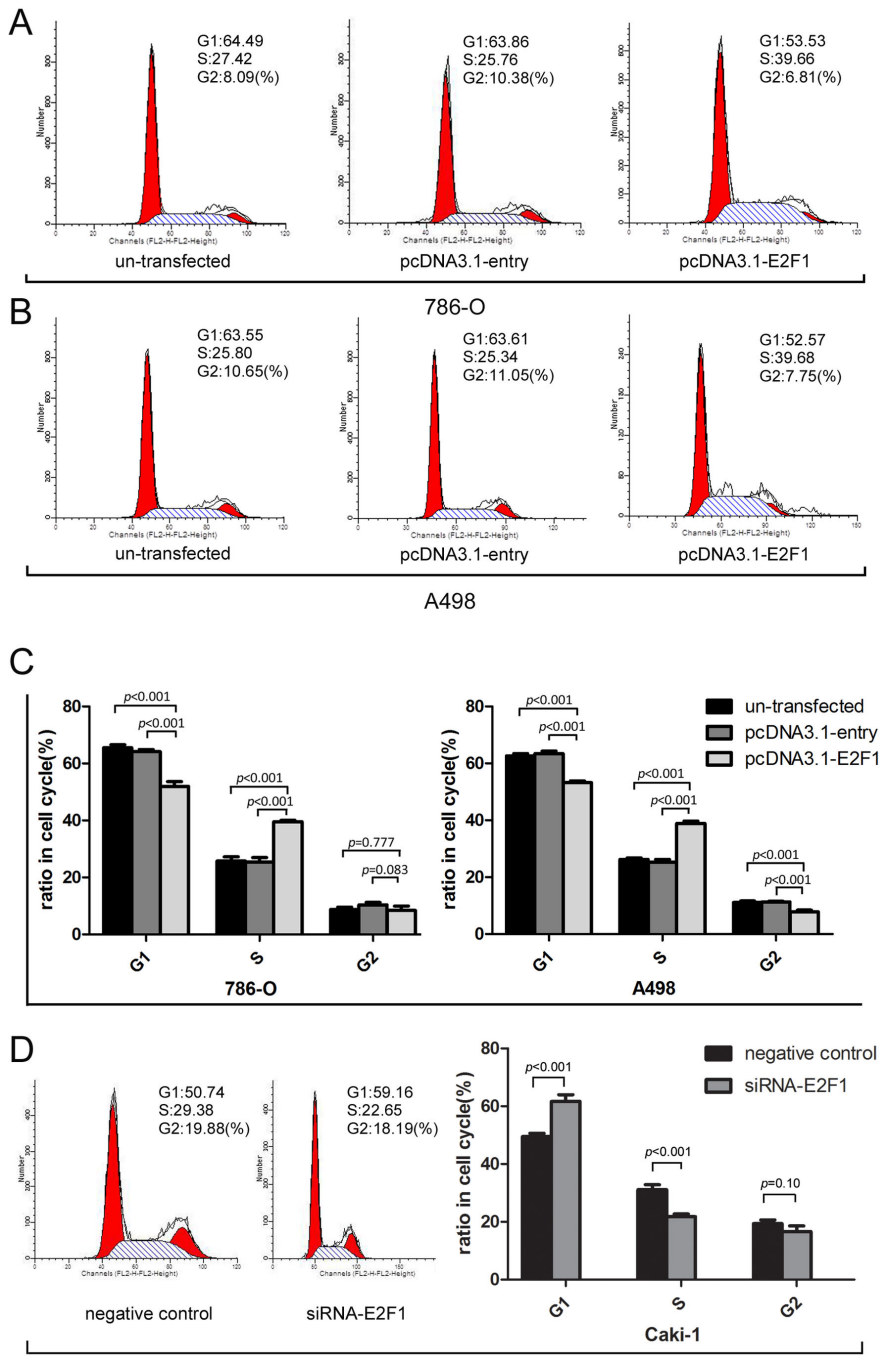
Cell cycle analysis of E2F1 interferences in 786-O, A498 and Caki-1 cells. (A) Representative photographs were chosen to show differences in groups of 786-O.The data were shown mean±standard deviation (SD). (B) Representative photograph illustrated the distinguished results in groups of cell line A498.The data were shown mean±SD. (C) E2F1 inducing G1-S phase transition in E2F1 group compared with un-transfected and entry group both in 786-O and A498 cells(*p*<0.001). (D) Knockdown of E2F1 induced G1 restoration compared with negative control group in Caki-1 cells (*p*<0.001). Representative experiments of three with similar results.

Flow cytometry analysis indicated that the overexpression of E2F1 protein markedly changed cell cycle distribution of A-498 and 786-O cells (*p*<0.001). (Figure 4A–C). For 786-O cells, the G1 ratio in E2F1 plasmid group was 51.97±1.66% compared with 65.47±1.11% in un-transfected group and 64.20±0.71% in entry group respectively(*p*<0.001) (Figure 4A). For A498 cells, the G1 ratio in E2F1 plasmid group was 53.22±0.58% versus 62.65±0.79% in un-transfected group and 63.47±0.86% in entry group respectively(*p*<0.001) (Figure 4B). Signiﬁcant differences were observed in the fraction of cells in G1 and S phase. Cell cycle analysis revealed that 786-O and A-498 cells rapidly gone through G1/S checkpoint 48 h after E2F1 plasmid transfection (Figure 4C). In contrast, knockdown of E2F1 restored Caki-1 cells in G1 phase (Figure 4D). These results suggested that E2F1 prompted the entry of cell cycle into S phase as previously reported. Therefore, E2F1 exerted an enforced effect on cell cycle progression which may partly explain the proliferation effect of E2F1 on ccRCC cells.

### Overexpression of E2F1 enhanced cell migratory and invasive ability of ccRCC in vitro.

**Figure 5:**
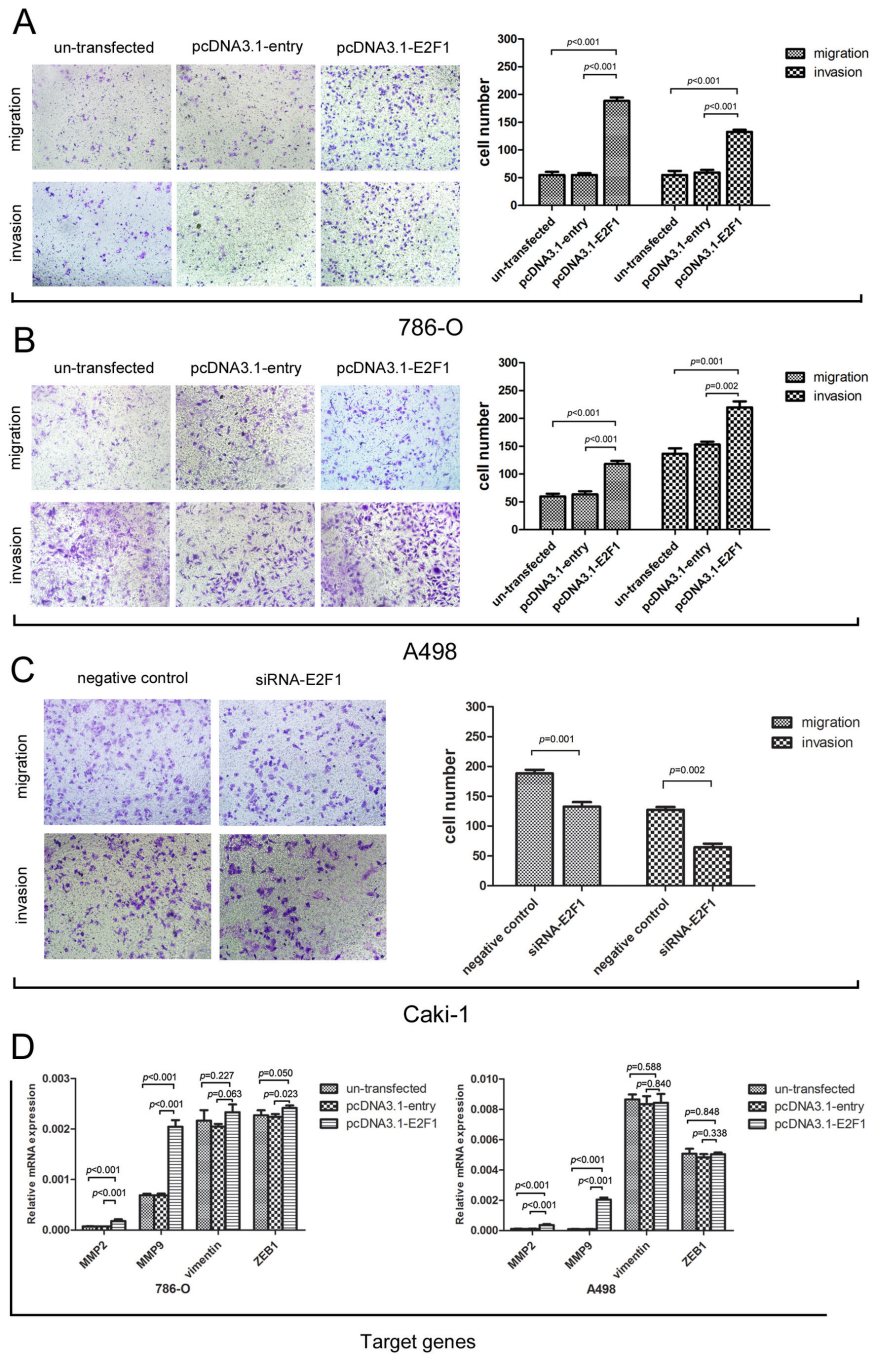
Effects of E2F1 in migration and invasion of cancer cells. (A) Representative view of 786-O migration and invasion transfected with E2F1 plasmid, entry plasmid and nothing blank respectively. (B) Representative view of A498 migration and invasion transfected with E2F1 plasmid, entry plasmid and nothing blank respectively. (C) Representative view of Caki-1 migration and invasion transfected with E2F1 siRNA sequence and negative control. (D) MMP2, MMP9, vimentin and ZEB1 were validated in three groups as aggressiveness related genes. MMP2, MMP9 mRNA expressions in E2F1 group were significantly elevated compared with untransfected and entry groups in 786-O and A498 cells. The data shown were mean±SD. Each Experiment was done in triplicate.

**Figure 6:**
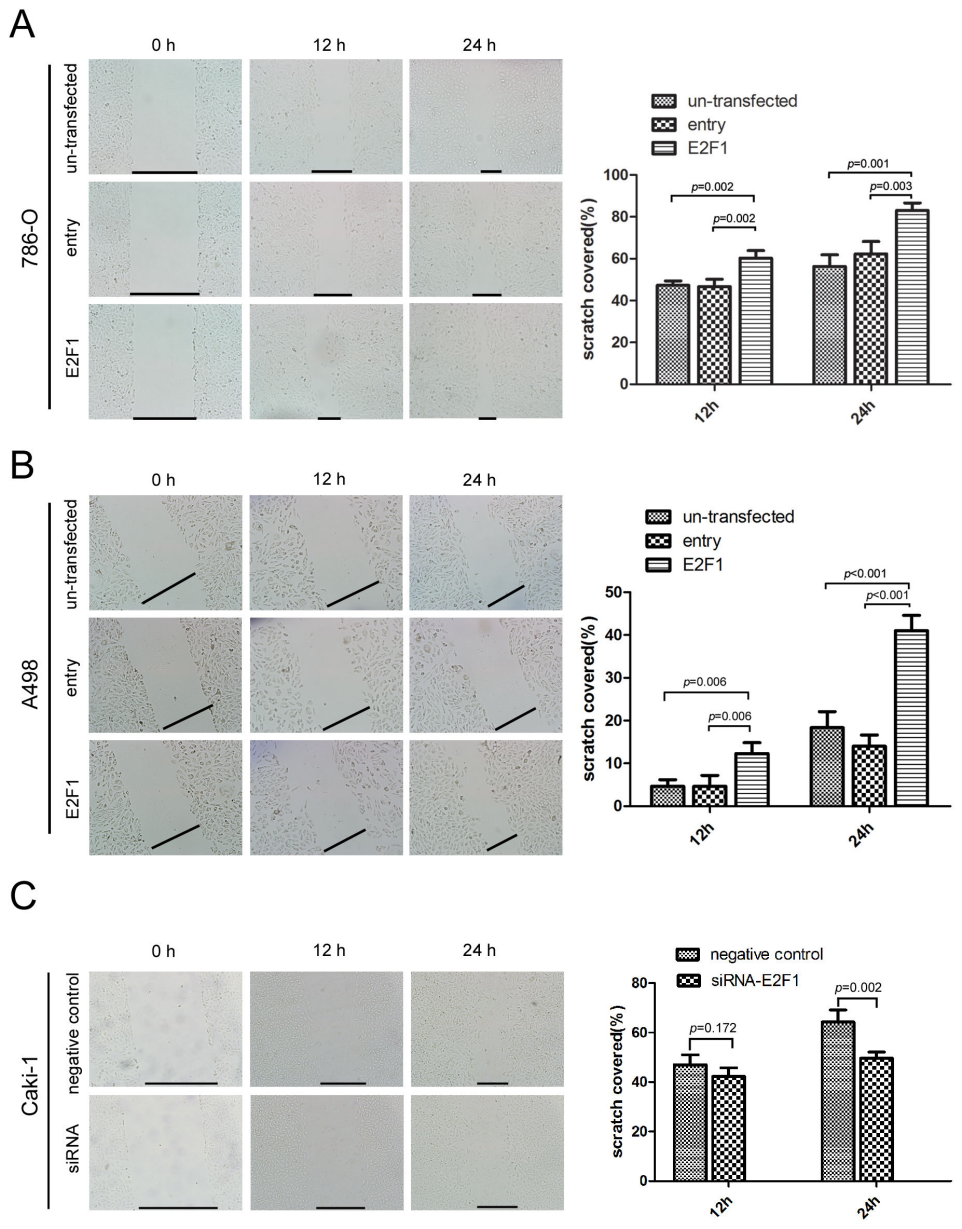
Effects of E2F1 on the abilities of in vitro cell motility and migration. Conﬂuent monolayer cells were scraped with a plastic 200 µl pipette tip. After replacing the cell culture medium, the wound closure was photographed at different time points (0, 12 ,24h after scraping). In 100 times microscope, areas of scraping cells were counted. (A) in 786-O cells E2F1 overexpressing significantly increased the number of viable cells (*p*<0.01). (B) In A498 cells E2F1 overexpressing largely promoted the cell viability (*p*<0.01). (C) In Caki-1 cells E2F1 knockdown markedly decreased the cell viability (*p*=0.002). Each Experiment was performed in triplicate.

Transwell experiments reflect the cell trans-membrane ability including migration and invasion. The effects on migration and invasion were observed 48 h after E2F1 plasmid transfection in 786-O and A498 cells using the Boyden chamber transwell assay. Trans-membrane cell numbers were counted and statistically analyzed. E2F1 plasmid transfected group showed a distinct result compared with the other two controls. For 786-O and A498 cells, E2F1 overexpression enforced more cells to penetrate the membrane (invasion assay with Matrigel) (*p*<0.001) (Figure 5A-B). With the same trend, E2F1 knockdown inhibited the Caki-1 migration and invasion(*p*=0.001) (Figure 5C). Given that MMP2, MMP9, vimentin, and ZEB1 were regarded as potential elemental genes implying the pro-metastatic state and higher malignancy of tumor cells, as well as E2F1 downstream target genes, RT-PCR was performed to detect changes in mRNA levels. Accordingly, MMP2 and MMP9 were found to be markedly upregulated after E2F1 plasmid interference (*p*<0.001) both in 786-O and A498, ZEB1 expression was elevated in 786-O (*p*=0.023). Therefore, we might infer that E2F1-inducing pro-metastatic potential and higher malignancy of ccRCC cell lines were probably dominated by vascular wall degradation related genes such as MMP2, MMP9 which functioned as cell-surface proteolysis of extra-cellular matrix components [16]. For wound healing assay, direct results of the motility of tumor cells were obtained as entering the intermediate empty space reflecting migratory ability independent of proliferation. Investigation into the effects of E2F1 interference illustrated that E2F1 augmented cell migration at different time points in 786-O (*p*<0.01, Figure 6A), A498 (*p*<0.01, Figure 6B) and Caki-1(*p*=0.002, Figure 6C) cells.

## Discussion

E2F1 plays a pivotal role in the nucleus as a transcription factor in charge of thousands of downstream target genes, thus loads of biological processes smoothly perform and function well [17]. Early studies have shown that E2F1 expression can be involved in tumor progression. Ju-Seog Lee et al reported that the expression signature of E2F1 and its associated genes predicted superﬁcial to invasive progression of bladder tumors [8]. In another study, E2F1 may participate in telomerase activity regulation in malignant glioma cells and its expression seemed to be strongly associated with the survival of patients with malignant brain tumors [18]. In a study that aimed to predict the outcome in Korean women who underwent surgery for breast carcinoma, the E2F1-positive group had less tumor recurrences, lymph node metastasis during follow-up, and distant metastasis than the E2F1-negative group [19]. In adenocarcinomas of Barrett esophagus E2F-1 overexpression was identified to be correlated with decreased proliferation and better prognosis [20]. These contradictory biological results exhibited by E2F1 probably depended on the tissue specificity. To further conﬁrm the role of E2F1 in ccRCC malignant progression, we employed E2F1 plasmid transfection technology to increase the expression of E2F1 gene in non-metastatic ccRCC cell lines 786-O and A498 with relatively low expression of E2F1 protein and RNAi knockdown of E2F1 in metastatic cell line Caki-1.

In the current study, we demonstrated that E2F1 was up regulated in ccRCC compared with corresponding peritumoral tissues both in mRNA and protein levels. E2F1 was mainly located in the nucleus of cancer cells, whereas in kidney proximal tubule cells cytoplasm and membrane were abundantly immunohistochemically stained.

As expected, the investigation into the effect of E2F1 overexpression illustrated that E2F1 exerted its apparent ability to intrigue the cell cycle and may be involved in carcinogenesis in ccRCC cell lines. Cell cycle analysis revealed that E2F1 overexpression cells possessed lower levels of G1 phase and higher S phase than controls. In agreement with previous documents, E2F1-induced G1/S transition [21] triggered tumor cell growth and colony forming which were consistent with larger tumor and higher pT grading. To explore the role of E2F1 in ccRCC expression we lay stress on analysis of clinicopathological parameters in patients. E2F1 mRNA levels directly correlated with tumor diameter, Fuhrman tumor grade, pT stage, TNM stage grouping and macravascular invasion (MAVI) excluding age and sex. Elevated E2F1 expression was associated with larger tumor size, advanced pT stage and higher TNM stage. This finding can be explained by the effect of E2F1 on tumor cell growth. Importantly, pT4 grade Tumor which invades beyond Gerota’s fascia (including contiguous extension into the ipsilateral adrenal gland) [2] needed to be emphasized. Given the statistical results we inferred that E2F1 may help cancer cell to penetrate Gerota’s fascia which constricted tumors locally, namely, metastatic process was evaluated. Of note, a more interesting ﬁnding was the signiﬁcant association of the enhanced E2F1 expression with the vascular inﬁltration of ccRCC (MAVI) embracing renal vein and inferior *vena* *cava* [4]. Hematogenous metastasis triggering is an appreciable incident suggesting higher malignancy and poorer clinical outcome. Angiogenic activity is elucidated to be a key stone in vascular invasion. MMPs take effects in the late events in the metastatic process and are principally vital in angiogenesis. Montesano et al reported that PMA-treated bovine microvascular endothelial cells which invaded the underlying collagen matrix can be hampered by the MMP inhibitor, 1,10-phenanthroline [22]. MMP2 and MMP9 are involved in intravasation by participating in extracellular matrix (ECM) conversion and remodeling through hydrolyzing major protein components when cancer cells must exit the blood vessels, lymphatics [16] and constricted capsules. We also highlighted vimentin and ZEB1; two Epithelial-to-Mesenchymal Transition (EMT) related genes. Vimentin directly mediates cell motility and thus leads to late stage carcinogenesis [23]. ZEB1, possessing EMT-related similar E-box sequence motifs, represses E-cadherin expression [24]. Similarly our findings shed light on cancer cell lines demonstrating that E2F1 overexpression significantly enforced the capacity of cell migration and invasion, thereby enriching the understanding of this versatile transcription factor independent of apoptotic activity and proliferation trait. In vitro, we found that MMP2 and MMP9 manifested great alterations after E2F1 plasmid transfection in both ccRCC cell lines, ZEB1 mRNA expression was elevated in 786-O only with the function to be confirmed. Owing to above results we concluded that tumor cells obtained migratory and invasive ability indicated by MMP2 and MMP9 stimulation, whereas ZEB1 required further elucidation in other ccRCC cell lines [25,26]. Another mechanism of E2F1-induced invasiveness has been confirmed by the E2F1-epidermal growth factor receptor interaction with E2F1 in melanoma progression [10]. However, in another study endogenous E2F1 was validated to attenuate angiogenic activity via p53-dependent transcriptional control of VEGF expression in vivo [20]. It appeared to be a paradox of vascular invasion. Moreover, whether other mechanisms exist remains to be explored.

In conclusion, E2F1 activation mediated higher malignancy and vascular infiltration potential in ccRCC by promoting the proliferation, migration and invasion capacities of cancer cells. MMP2 and MMP9 were identified to exert crucial effect as the indicators in cancer progression. Currently no efﬁcient treatment is available for advanced or even metastatic ccRCC, apart from using some kinase inhibitors such as sorafenib and sunitinib which performed preferential effects on progression-free survival for patients with high grade ccRCC [27]. Thus aberrant expression of E2F1 and its correlation with clinicopathological features may provide new insights into the improvement of novel clinical therapeutics.
